# Effects of Qutan Huoxue Formula on the SOCS1/TLR4 Signaling Pathway in NASH Model Mice

**DOI:** 10.1155/2020/1570918

**Published:** 2020-11-20

**Authors:** Yurong Zhang, Xiaoning Zhu, Ding Zheng, Yue Yin, Mengyun Peng, Jing Wang

**Affiliations:** Affiliated Traditional Chinese Medicine Hospital of Southwest Medical University, Luzhou 646000, China

## Abstract

The purpose of this study was to investigate the effects of Qutan Huoxue Formula (QHF) on liver injury in mice with nonalcoholic steatohepatitis (NASH) by upregulating SOCS1 to inhibit the TLR4/NF-*κ*B signaling pathway. Thirty male C57BL/6J mice (20–22 g) were randomly divided into the normal diet group (ND group), methionine- and choline-deficient diet group (MCD group), and Qutan Huoxue Formula group (QHF group). Mice in the ND group were fed a regular diet, while mice in other two groups were fed MCD diet. After successful molding, the QHF group was gavaged by QHF. The ND group and MCD group were gavaged by the same volume of normal saline, once a day. During the period of gavaging, all mice continue to be fed MCD fodder except for the ND group. All mice were killed at 8 w. H&E staining and Oil Red O staining were used to observe the pathological changes of liver tissues. Serum level of ALT, AST, TC, and TG was detected by enzyme-linked immunosorbent assay. The expression of liver SOCS1, TLR4, Myd88, and NF-*κ*B was detected by real-time PCR, immunohistochemistry, and Western blot. QHF can significantly reduce the serum levels of ALT, AST, TC, and TG of NASH mice and reduce the degree of liver fat degeneration and inflammation. It also can decrease both mRNA and protein expressions of liver TLR4, Myd88, and NF-*κ*B. The mRNA expression of SOCS1 increased, while the SOCS1 protein expression decreased. In conclusion, QHF can significantly alleviate hepatic steatosis and inflammation in NASH mice by upregulating SOCS1 to inhibit the TLR4/NF-*κ*B signaling pathway.

## 1. Introduction

Nonalcoholic fatty liver disease (NAFLD) has developed into the most common chronic liver disease in the world. The disease spectrum includes nonalcoholic fatty liver (NAFL), nonalcoholic steatohepatitis (NASH), related cirrhosis, and hepatocellular carcinoma (HCC) [1, 2]. NASH is considered to be the most important part in the development of NAFL into cirrhosis and HCC. Its occurrence is mainly due to the abnormal increase of free fatty acids (FFAs) in the circulation, which exceeds the liver's oxidative and metabolic capabilities. Lipids, which deposited in hepatocytes, induced that a large amount of peroxides are produced, and then, Kupffer cells are activated through upgraduating Toll-like receptor 4 (TLR4) signaling by the Myd88-dependent pathway. Thereby, activated Kupffer cells initiate the NF-*κ*B pathway to induce apoptosis in hepatocytes [3–6]. Studies have shown that overexpression of the suppressor of cytokine signaling 1 (SOCS1) can directly inhibit the activation of the TLR4/NF-*κ*B signaling pathway to improve liver inflammation [7].

QHF is an experience formula for treating NASH under the guidance of the “Harmony-Reconciling Shaoyang” ideology. Previous studies have confirmed [8, 9] that QHF can significantly reduce the serum levels of ALT and AST in patients with NASH, and its mechanism is related to the reduction of NF-*κ*B expression. However, further research is needed to explore how QHF regulates NF-*κ*B expression to treat NASH. Therefore, this study focused on observing the effects of QHF on improving liver injury in NASH by upregulating the expression of SOCS1 to inhibit the TLR4/NF-*κ*B signaling pathway.

## 2. Materials and Methods

### 2.1. Materials

#### 2.1.1. Experimental Animals

Thirty SPF male C57BL/6J mice, weighing 20–22 g, were purchased from Chengdu Dossy Experimental Animals, Co., Ltd. (SCXK (Sichuan), 2015–030). All mice were kept at 20±2°C with a 12 h light/dark cycle under specific pathogen-free conditions. The experiment has been approved by the Ethics Committee of Southwest Medical University.

#### 2.1.2. Experimental Drugs and Reagents

Experimental drugs: MCD diet (purchased from Trophic Animal Feed High-tech Co., Ltd., China, TP3006R); QHF extract preparation: the composition of QHF is shown in Table 1. The dose conversion is calculated according to the conversion ratio of the surface area between humans and animals. In this study, the optimal therapeutic dose was based on the previous research results [10]. The decoction, extraction, and concentration of Chinese medicinal materials are completed by the preparation department of the hospital of traditional Chinese medicine affiliated to Southwest Medical University.

Reagents: ALT, AST, TC, and TG kits (Nanjing Jiancheng bioengineering institute, C009-2, C010-2, A111-1, and A110-1), Oil Red O staining kit (Solarbio, G1261-2), total RNA extraction kit (Tiangen Biotech (Beijing) Co., Ltd., DP419), reverse transcription kit, and SYBR Green Real-time PCR Master Mix (Toyobo, QPS-201). The primer sequences were synthesized by Sangon Biotech Co., Ltd. (Shanghai); TLR4, SOCS1, and NF-*κ*B antibodies (Abcam, ab13556, ab62584, and ab16502), and Myd88 (Santa, sc-74532).

#### 2.1.3. Main Instruments and Equipment

Ultra-low temperature refrigerator (Thermo), automatic microplate reader (Finnpipette Co., Ltd.), fluorescence microscope (Nikon), high-speed low temperature centrifuge (Sigma), real-time PCR instrument (Eppendorf, Germany), and gel scanning imaging system (Bio-Bad, USA).

### 2.2. Methods

#### 2.2.1. Grouping and Modeling

After one week of adaptive feeding, thirty C57BL/6J mice were randomly divided into the ND group (ND), MCD group (MCD), and QHF group (QHF). The ND group was fed normal diet, and the MCD group and QHF group were fed MCD diet. At the end of the 4th week, two mice were randomly selected from the MCD group and QHF group, respectively, for H&E staining to determine the success of modeling. After that, mice in the QHF group were gavaged with QHF, while mice in other two groups were gavaged with equal volume of normal saline for 4 weeks. During total experiment, mice both in the MCD group and QHF group were fed MCD continuously.

#### 2.2.2. General Condition and Weight of Mice

Mice weighed, feces, and external reactions were recorded weekly.

#### 2.2.3. Biochemical Detection

The concentration of serum TC, TG, ALT, and AST was determined by enzyme-linked immunosorbent assay.

#### 2.2.4. Histopathology and Immunohistochemistry

Fresh tissue was fixed and dehydrated. Liver steatosis and inflammation were observed by H&E and Oil Red O staining. The expression of SOCS1, TLR4, Myd88, and NF-*κ*B was detected by immunohistochemistry.

#### 2.2.5. Real-Time PCR

Hepatic tissue total RNA was extracted and reverse-transcribed into cDNA. Real-time PCR was performed according to the kit instructions. *β*-Actin was used as an internal reference. The expression of relative target gene mRNA was performed using a comparative cycle threshold (CT) method. The primer sequences are shown in Table 2.

#### 2.2.6. Western Blot

The liver tissue was ground with liquid nitrogen and lysed on ice for 30 minutes. Lysates were separated by SDS-PAGE and transferred onto PVDF membranes. After blocking and incubating with primary antibodies, these blots were incubated with anti-rabbit or anti-mouse secondary antibodies. Protein bands were detected using an enhanced chemiluminescence system and analyzed with Image J software.

#### 2.2.7. Statistical Analysis

All data were analyzed using SPSS 23.0 and graphed with GraphPad Prism8.0. Values in the text are means ± standard deviation ($\bar{x}\pm s$). The comparison among the three groups was performed by analysis of variance and pairwise comparison by the Shapiro–Wilk test. *P* < 0.05 was considered statistically significant.

## 3. Results

### 3.1. Effect of QHF on the General Condition and Weight Change of Mice

**Figure 1:**
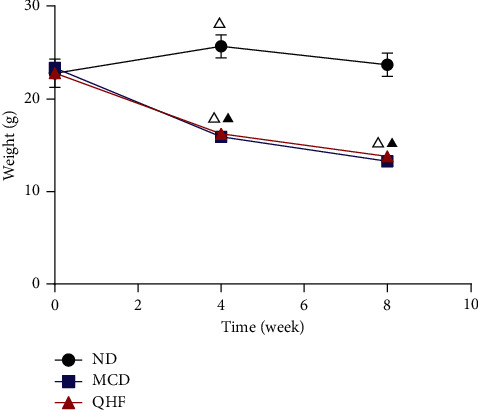
The effect of QHF on the weight change of mice. ^△^*P* < 0.05 vs. previous time point in the QHF group, ^▲^*P* < 0.05 vs. the ND group at the same time point.

During the experiment, feces of the three groups of mice were basically normal. The mice in the ND group had sensitive movements and shiny fur, while the mice in the MCD group had significant weight loss, less activity, and greasy fur. After the intervening of QHF for 4 weeks continuously, the general condition has been relieved. In the ND group, the body weight of mice were higher at week 4 than before (*P* < 0.05), and the body weight of mice at week 8 and week 4 were similar (*P* > 0.05). The body weight of the mice in the MCD group and the QHF group gradually decreased with time (*P* < 0.05). After intervening, the downward trend of mice body weight in the QHF group at week 8 was slower than that at week 4. There was no body weight difference between the QHF group and the MCD group at week 8 (*P* > 0.05) (Figure 1).

### 3.2. Effect of QHF on Serum Levels of ALT, AST, TC, and TG

**Figure 2:**
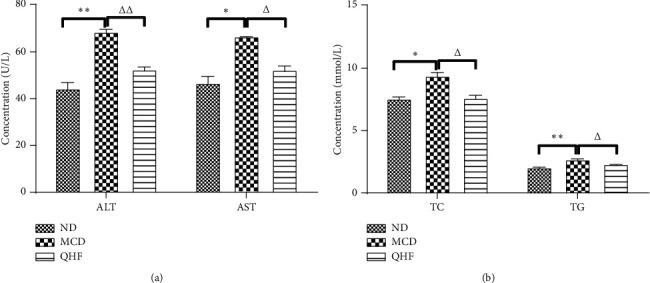
The effect of QHF on serum levels of ALT, AST, TC, and TG. ALT, alanine aminotransferase; AST, aspartate transaminase; TC, total cholesterol; TG, triglyceride. ^*∗*^*P* < 0.05, ^*∗∗*^*P* < 0.01 vs. the ND group; ^△^*P* < 0.05, ^△△^*P* < 0.01 vs. the MCD group.

The serum levels of ALT, AST, TC, and TG in the MCD group were higher than that in the ND group, and those in the QHF group was lower than that in the MCD group. These results indicated that QHF can improve the serum liver function and blood lipid levels in NASH mice (Figures 2(a) and 2(b)).

### 3.3. Effect of QHF on Liver Pathological Changes of NASH Mice

**Figure 3:**
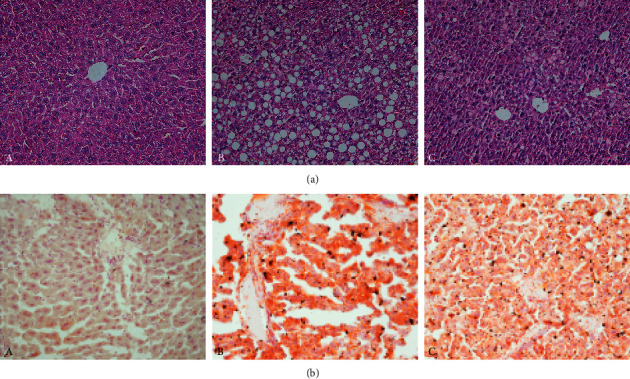
The effect of QHF on liver pathological changes of NASH mice. (a) H&E staining (×200). (b) Oil Red O staining (×200). A, ND group. B, MCD group. C, QHF group.

H&E staining: in the ND group, the hepatic lobule structure was complete, and the hepatocytes were neatly arranged. In the MCD group, the hepatic lobule structure of mice was damaged. A large number of balloon-like cells and infiltration with inflammatory cells were observed in hepatocytes. Compared to the MCD group, the degree of inflammation and lipoidosis in hepatocytes of mice in the QHF group was significantly improved after 4 weeks of treatment (Figure 3(a)).

Oil Red O staining: abundant lipid droplets were observed in hepatocytes of mice in the MCD group, and compared with the MCD group, the lipid accumulation was significantly ameliorated after QHF treatment (Figure 3(b)).

### 3.4. Effect of QHF on SOCS1, TLR4, Myd88, and NF-*κ*B Expression

**Figure 4:**
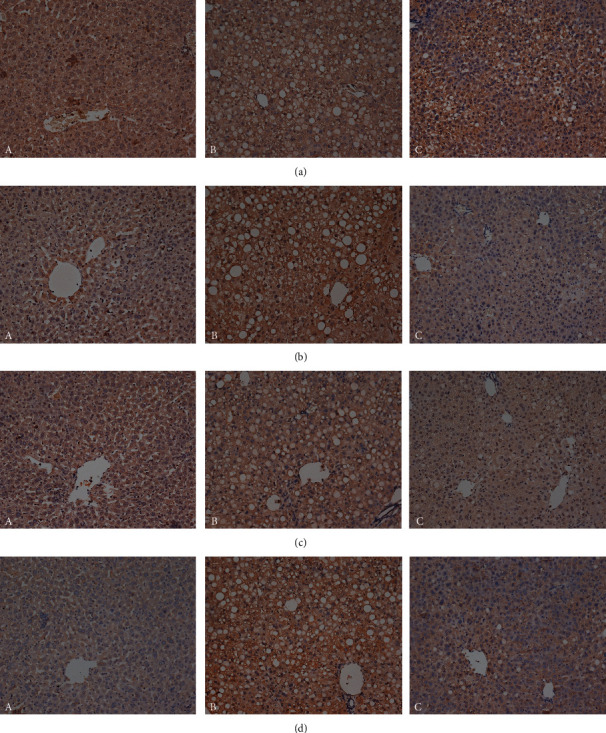
(a–d) The effect of QHF on SOCS1, TLR4, Myd88, and NF-*κ*B expression (×200). A, ND group. B, MCD group. C, QHF group.

Compared with the MCD group, mice in the QHF group showed less positive expression of TLR4, Myd88, and NF-*κ*B and higher positive expression of SOCS1 in liver sections (Figures 4(a)–4(d)).

### 3.5. Effect of QHF on SOCS1, TLR4, Myd88, and NF-*κ*B mRNA Expression

**Figure 5:**
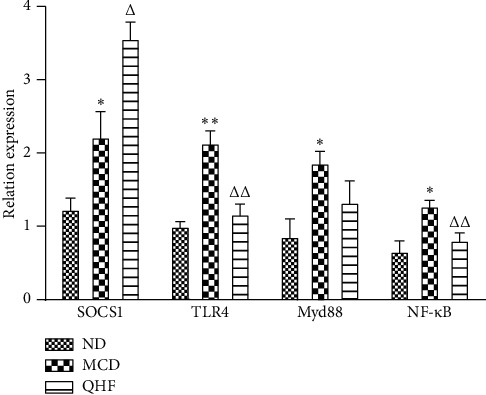
The effect of QHF on SOCS1, TLR4, Myd88, and NF-*κ*B mRNA expression. ^*∗*^*P* < 0.05, ^*∗∗*^*P* < 0.01 vs. the ND group; ^△^*P* < 0.05, ^△△^*P* < 0.01 vs. the MCD group.

Compared with the ND group, the expression levels of SOCS1, TLR4, Myd88, and NF-*κ*B in the liver tissues of the MCD group were higher (*P* < 0.05). Compared with the MCD group, the expression levels of TLR4, Myd88, and NF-*κ*B in liver tissues of mice in the QHF group were lower (*P* < 0.05), while the expression of SOCS1 increased (*P* < 0.05) (Figure 5).

### 3.6. Effect of QHF on SOCS1, TLR4, Myd88, and NF-*κ*B Protein Expression

**Figure 6:**
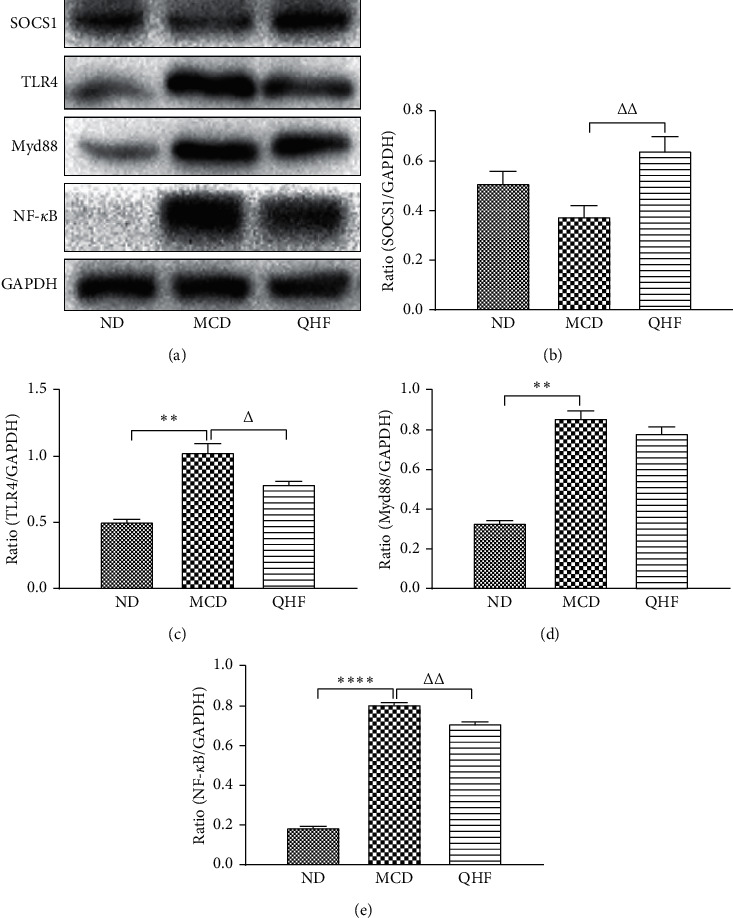
The effect of QHF on SOCS1, TLR4, Myd88, and NF-*κ*B protein expression. ^*∗*^*P* < 0.05, ^*∗∗*^*P* < 0.01, ^*∗∗∗∗*^*P* < 0.0001 vs. the ND group; ^△^*P* < 0.05, ^△△^*P* < 0.01 vs. the MCD group.

Compared with the MCD group, QHF significantly decreased the protein expression of TLR4, Myd88, and NF-*κ*B and increased the protein expression of SOCS1 in NASH mice (Figure 6).

## 4. Discussion

The pathogenesis of NASH is closely related to various factors such as excessive accumulation of lipids in hepatocytes, oxidative stress, mitochondrial damage, and activation of Kupffer cells. The activation of Kupffer cells activated by the TLR4/NF-*κ*B pathway is one of the key factors for liver injury in NASH [3, 11]. Recent studies [12, 13] have found that both the abnormal accumulation of lipids in hepatocytes and the abnormally increased LPS can activate the TLR4/NF-*κ*B pathway to induced Kupffer cells activated. Thereby, releasing a large number of proinflammatory factors cause liver injury, which ultimately leads to NASH. In this study, we copied the MCD diet-induced NASH mouse model to observe the effect of QHF on NASH liver injury. We found that the mice body weight in the MCD group was significantly reduced. The liver pathology showed a large number of lipid droplets, balloon-like changes, and inflammatory cell infiltration. The serum levels of ALT, AST, TC, TG, and mRNA and protein expressions of TLR4, Myd88, and NF-*κ*B in liver tissues were also significantly increased. It demonstrated that mice had severe liver damage due to excessive accumulation of hepatocyte lipids. After intervention of the Chinese herbal medicine, the levels of liver inflammation and fat change and serum ALT, AST, TC, TG, and mRNA and protein expression levels of TLR4, Myd88, and NF-*κ*B in the QHF group were significantly reduced. This decrease indicates that QHF can improve liver injury in NASH mice through inhibiting the TLR4/NF-*κ*B pathway.

SOCS1, which belongs to the family of cytokine signaling inhibitors, is a class of negative regulators produced by cells and blocking the cytokine signal transduction process in feedback. Current research [14] has shown that after the activation of Kupffer cells, the increased proinflammatory factors will upregulate SOCS1 expression, and thus inhibit TLR4/NF-κB signaling pathway to protect hepatocytes. Currently, there are two views on the action of SOCS1 with the TLR4/NF-*κ*B signaling pathway [6]. One view holds that SOCS1 directly interferes TLR4/NF-*κ*B signaling molecules. The other view holds that SOCS1 directly or indirectly interferes the TLR4/NF-*κ*B signal transduction pathway by acting on signal molecules with different sensitivities. The results of this study showed that the mRNA and protein expression of TLR4, Myd88, and NF-*κ*B in the MCD group increased, while the mRNA expression of SOCS1 increased and the protein expression decreased. After gavaging with the Chinese herbal medicine, the mRNA and protein expression of TLR4, Myd88, and NF-*κ*B in the QHF group reduced, while the mRNA and protein expression of SOCS1 increased. The above results indicate that QHF can increase the expression of SOCS1 and directly inhibit the activation of the TLR4/NF-*κ*B signaling pathway. In addition, we hypothesized that the inconsistent expression of mRNA and protein of SOCS1 caused by liver injury was related to the enhanced transcriptional inhibition of genes.

Professor Sun Tongjiao considered that phlegm and dampness developing into blood stasis are the starting factors for the onset of NASH, phlegm being adhesive in nature and dampness being wet and cold. The stasis of the two is most likely to block water passage and damage Yang qi. The gallbladder is the hinge of qi regulation and governs ministerial fire. The triple energizer regulates water passage and ministerial fire fumigation. Qi and water disseminated to all parts of the body. Yang qi operation and communication between inside and outside is called Shaoyang pivot. Phlegm and blood stasis were produced by dysfunction of Shaoyang pivot, and then, phlegm and blood stasis interaction will affect the dispersing and dredging function of the liver. Therefore, the dysfunction of Shaoyang pivot is considered to be the key pathogenesis; so, the QHF was used to treat NASH. The QHF consists of tangerine peel, Poria, and Rhizoma Pinelliae Preparata. All herbs used together play the functions of harmonizing Shaoyang, agitating the qi movement of the middle energizer and promoting blood circulation and dehumidification. Relevant research has shown that [15, 16] Radix Bupleuri, Radix Scutellariae, Radix Curcumae, and Radix Salviae Miltiorrhizae have the ability to inhibit the secretion of inflammatory mediators, scavenge free radicals, antioxidant, and to regulate immune functions. Tangerine peel, Poria, Rhizoma Pinelliae Preparata, Semen Coicis, and Rhizoma Alismatis can regulate lipid metabolism. It can be seen that the effect of QHF in improving NASH liver injury is closely related to the reduction of excessive accumulation of lipid in hepatocytes, the inhibition of inflammation, and the regulation of immune balance.

In summary, the QHF has a clear effect on improving NASH liver injury, and its mechanism is related to upregulation of SOCS1 expression to inhibit the activation of the TLR4/NF-*κ*B signaling pathway. However, the mechanism of how QHF upregulates SOCS1 expression remains to be further explored.

## Figures and Tables

**Table 1:** The composition of Qutan Huoxue formula.

Chinese name	English name	Proportion (g)
Chenpi	Tangerine peel	10
Fulin	Poria	10
Fabanxia	Rhizoma Pinelliae Preparata	10
Yiyiren	Semen Coicis	20
Zexie	Rhizoma Alismatis	20
Yujin	Radix Curcumae	15
Danshen	Radix Salviae Miltiorrhizae	15
Shanzha	Chinese hawthorn fruit	15
Chaihu	Radix Bupleuri	12
Huangqin	Radix Scutellariae	10
Juemingzi	Semen Cassiae	15
Zhigancao	Radix Glycyrrhizae Preparata	3

**Table 2:** Primer sequence of real-time PCR.

Genes	Primer sequence	Amplified fragments
*β*-Actin F	5′-GGCTGTATTCCCCTCCATCG-3′	154 bp
*β*-Actin R	5′-CCAGTTGGTAACAATGCCATGT-3′	
Myd88 F	5′-TCATGTTCTCCATACCCTTGGT-3′	175 bp
Myd88 R	5′-AAACTGCGAGTGGGGTCAG-3′	
TLR4 F	5′-GCCATCATTATGAGTGCCAATT-3′	107 bp
TLR4 R	5′-AGGGATAAGAACGCTGAGAATT-3′	
SOCS1 F	5′-CTGCGGCTTCTATTGGGGAC-3′	216 bp
SOCS1 R	5′-AAAAGGCAGTCGAAGGTCTCG-3′	
NF-*κ*B F	5′-ATGGCAGACGATGATCCCTAC-3′	111 bp
NF-*κ*B R	5′-TGTTGACAGTGGTATTTCTGGTG-3′	

## Data Availability

The data used to support the findings of this study are available from the corresponding author upon request.
